# Anti-TNF Therapies Promote a Proximal-to-Distal Healing Pattern in Moderate-to-Severe Ulcerative Colitis

**DOI:** 10.1093/ibd/izaf199

**Published:** 2025-09-25

**Authors:** Emily C L Wong, Parambir S Dulai, John K Marshall, Vipul Jairath, Walter Reinisch, Neeraj Narula

**Affiliations:** Division of Gastroenterology, Department of Medicine and Farncombe Family Digestive Health Research Institute, McMaster University, Hamilton, ON, Canada; Division of Gastroenterology, Northwestern University, Chicago, IL, United States; Division of Gastroenterology, Department of Medicine and Farncombe Family Digestive Health Research Institute, McMaster University, Hamilton, ON, Canada; Division of Gastroenterology, Department of Medicine, Western University, London, ON, Canada; Division of Gastroenterology and Hepatology, Department of Internal Medicine III, Medical University of Vienna, Vienna, Austria; Division of Gastroenterology, Department of Medicine and Farncombe Family Digestive Health Research Institute, McMaster University, Hamilton, ON, Canada

**Keywords:** ulcerative colitis, Mayo endoscopic score, endoscopic improvement

## Abstract

**Background:**

Ulcerative colitis (UC) is a chronic inflammatory disease of the colonic mucosa, extending proximally from the rectum. However, the segmental pattern of healing in UC remains unclear. Endoscopic improvement (EI), a key therapeutic endpoint, is typically assessed using the Mayo endoscopic score (MES), which scores the worst affected area and may miss partial/segmental healing. This study evaluates healing patterns in UC and compares conventional MES with a 3-segment MES approach for detecting treatment effects in clinical trials.

**Methods:**

A post hoc analysis of HIBISCUS I/II and GARDENIA trials was conducted in UC patients with moderate-to-severe disease (MES >2 up to the descending colon). The primary outcome was the proportion of anti-tumor necrosis factor–treated participants achieving MES ≤1 in the descending colon, sigmoid colon, and rectum at week 10. Secondary outcomes included conventionally measured EI, segmental MES improvements, clinical response, and Patient-Reported Outcome 2 (PRO2) normalization. Outcomes were compared between adalimumab, infliximab, and placebo groups.

**Results:**

Among 300 participants, 217 received infliximab or adalimumab, while 83 received placebo. Healing followed a proximal-to-distal pattern, with the highest EI in the descending colon and the lowest in the rectum. Infliximab-treated patients continued this trend at week 54. Anti-tumor necrosis factor therapy significantly improved EI vs placebo (42.9% vs 19.3%; *P* < .001). No segmental MES approach outperformed conventional MES for detecting treatment effects. Combined endpoints (MES ≤1 + PRO2 normalization) better captured therapeutic benefits than PRO2 alone (28.6% vs 13.3%; *P* = .006).

**Conclusions:**

UC healing follows a proximal-to-distal pattern. Conventional MES remains superior for detecting treatment effects over segmental MES. Further studies should explore alternative endoscopic scoring methodologies.

Key Messages *What is already known?*

- Ulcerative colitis (UC) typically extends in a continuous manner from the rectum proximally.
- Endoscopic improvement (EI), measured by the Mayo endoscopic score (MES), is a validated and widely used endpoint in UC clinical trials.
- Healing of the colonic mucosa is associated with better long-term clinical outcomes, including reduced risk of relapse and need for colectomy.

*What is new here?*

- This study is the first to demonstrate a proximal-to-distal healing pattern in UC using centralized endoscopic assessments.
- Despite limitations in granularity, the conventional MES remains the most effective metric for detecting treatment effects over more complex segmental scoring systems.
- Combining endoscopic and symptomatic endpoints (MES ≤1 + Patient-Reported Outcome 2 normalization) provides better discrimination of treatment response compared with Patient-Reported Outcome 2 alone.

*How can this study help patient care?*

- Highlights that persistent rectal inflammation at early follow-up may not indicate treatment failure if proximal healing is evident—this could inform timing of therapeutic decisions.
- Supports the continued use of conventional MES as a practical and sensitive endpoint in both trials and clinical practice.
- Suggests that integrating objective endoscopic data with patient-reported outcomes improves evaluation of therapeutic response and may guide personalized treatment strategies.

## Introduction

Ulcerative colitis (UC) is a chronic inflammatory disease of the colon, characterized by continuous mucosal inflammation that progresses proximally from the rectum.^1^ The pattern in which UC heals is unclear. The assessment of therapeutic efficacy in UC relies on endoscopic scoring systems, which quantify the severity of inflammation and degree of mucosal healing. Among these scoring systems, the Mayo endoscopic score (MES) is widely used in clinical trials and practice. Endoscopic improvement (EI), conventionally defined as MES ≤1, is an important endpoint for therapies for UC, as EI is associated with a reduced need for colectomy, hospitalization and relapse risk.^2–6^

The importance of EI has been emphasized by STRIDE (Selecting Therapeutic Targets in Inflammatory Bowel Disease) initiative of the International Organization for the Study of Inflammatory Bowel Diseases, with guidelines suggesting assessment within 3 to 6 months after starting therapy.^7^ However, the ability of the MES to capture total endoscopic inflammatory burden may be limited by its lack of granularity, particularly at the segmental level, as the MES is scored from 0 (normal or inactive disease) and 3 (severe disease) based on the worst-affected area of the colon.^8^ There is concern that within clinical trials, use of the conventional MES could limit the ability to detect treatment effect. For instance, a patient who had severe endoscopic disease throughout their colon and was scored MES 3 who achieved healing of most of their colon after initiation of active therapy but still had persistent rectal ulceration would continue to score an MES 3, despite improvement in the remainder of the colon. Given this limitation, there is growing interest in assessing segmental EI in UC.^9^^,^^10^

In this study, our aim was to elucidate patterns of healing in moderate-severe UC. Further, we aimed to assess whether a segmental endoscopic scoring approach in patients with moderate-severely active disease up to the descending colon is better able to detect treatment effect compared with the conventionally used worst-affected area as scored by the MES. Additionally, we investigate the extent to which improvements in specific segments correlate with overall clinical outcomes.

## Methods

### Data acquisition

This was a post hoc analysis of participant-level clinical trial data from HIBISCUS I (NCT02163759), HIBISCUS II (NCT02171429), and GARDENIA (NCT02136069). Data were obtained by permission from Roche Inc. and accessed through Vivli (protocol #00010343). Details regarding eligibility criteria and study methodology are detailed in previous publications.^11^^,^^12^ The Hamilton Integrated Research Ethics Board determined that a local ethics review was not necessary as previously deidentified data were used; therefore, no informed consent was required.

In short, HIBISCUS I and II were head-to-head induction trials evaluating etrolizumab, a gut-targeted anti-β7 integrin monoclonal antibody, against an active comparator, adalimumab, and placebo. Patients with moderate-severe UC (defined by a total Mayo score [MS] of 6-12 with an endoscopic subscore ≥2, rectal bleeding subscore [RBS] ≥1, and stool frequency subscore ≥1) and who were naïve to anti-tumor necrosis factor (TNF) agents were eligible. The primary endpoint was clinical remission at week 10 (MS ≤2, with no individual subscores >1, and RBS of 0). GARDENIA was a maintenance study evaluating etrolizumab against an active comparator, infliximab. Similar to HIBISCUS I and II, participants were eligible if they had moderate-severe UC (MS of 6-12 and an endoscopic subscore of ≥2, RBS ≥1, and stool frequency subscore ≥1). The primary endpoint was clinical response at week 10 (reduction in MS of at least 3 points and ≥30% reduction from baseline, in addition to a reduction of at least 1 point in RBS or absolute RBS of 0 or 1) and clinical remission at week 54 (MS ≤2 with no individual subscores >1). In all 3 studies, endoscopy (sigmoidoscopy or colonoscopy) occurred at baseline and week 10, and in the case of GARDENIA, also at week 54. All endoscopies were centrally read and scored using the MES. This study was a post hoc analysis that relied on the endoscopic scores provided by the source clinical trials. No independent re-reading or adjudication of the endoscopic assessments was performed. Data for anti-TNF– and placebo-treated patients were provided by Roche Inc. Data on etrolizumab-treated patients were not made available for this analysis.

### Participants

To demonstrate patterns of healing, we included participants with moderate-severely active disease (defined as MES 2 or 3) in the descending colon who had available endoscopic data at baseline and week 10. In all 3 studies, a total of 401 participants treated with anti-TNF or placebo had disease in the descending colon, of which 372 had week 10 scores available. Of these participants, a total of 300 had baseline MES 2 or 3 in the descending colon and were included in the current analysis.

### MES definitions

In this analysis, the MES scored out of 3 is referred to as “conventional MES.” The “segmental MES” was calculated as the sum of the conventional MES in each colonic segment assessed (ie, descending colon, sigmoid colon, and rectum), and was scored out of 9.

### Outcome definitions

The primary outcome of this study was to evaluate the proportions of anti-TNF–treated participants with moderate-severe UC who achieved MES ≤1 in the descending colon, sigmoid colon, and rectum after 10 weeks of active therapy. Long-term patterns of healing were assessed among infliximab-treated patients in GARDENIA, as this was the only included study with available endoscopic data at week 54. The main secondary outcome was to evaluate treatment effect between anti-TNF– and placebo-treated patients at week 10 using EI (defined as conventionally measured MES ≤1) and alternative definitions of endoscopic response assessed based on segmental (descending colon, sigmoid colon, rectum) MES improvements. Other secondary outcomes included clinical outcomes at week 10, including clinical response (reduction in total MS ≥3 points and ≥30% from baseline with an accompanying decrease in RBS of ≥1 point or absolute RBS of ≤1 point) and various definitions of Patient-Reported Outcome 2 (PRO2) normalization, which were selected to explore different measures of symptom improvement and to assess the additional benefit of PRO2 normalization. Sensitivity analyses were performed for clinical outcomes among patients with mild endoscopic disease at week 10 (MES ≤1). All outcomes were compared between anti-TNF–treated (adalimumab or infliximab) and placebo-treated participants. Sensitivity analyses were also performed to compare all outcomes between all 3 treatment groups individually (adalimumab vs infliximab vs placebo).

### Statistical analysis

Descriptive statistics were used to summarize the baseline characteristics of the study population. Categorical variables were reported as proportions or percentages and continuous variables were reported as mean ± SD or median (interquartile range). Chi-square tests and 1-way analysis of variance were used to compare the proportions of participants achieving outcomes. Outcomes at week 10 were compared between treatment groups. All analyses were performed on an intention-to-treat basis. All analyses were performed as intention-to-treat in the included population. A 2-sided *P* value <.05 was considered statistically significant. All analyses were performed using Stata IC version 18 (StataCorp).

## Results

### Baseline characteristics

Table 1 demonstrates the baseline characteristics of the study population. A total of 300 participants were included, of whom 144 were treated with adalimumab, 73 were treated with infliximab, and 83 received placebo. The mean age of participants was 38.8 ± 13.0 years, and 41.3% of participants were female. The average disease duration was 5.9 ± 6.3 years, and baseline endoscopic disease severity, as measured by the conventional MES (mean 2.5 ± 0.5) and segmental MES (mean 7.0 ± 1.5), was similar across groups. There were no significant differences in concomitant steroid use, concomitant immunomodulator use, C-reactive protein levels, RBS, or stool frequency subscore between groups. However, baseline fecal calprotectin levels were significantly higher in the infliximab group compared with the adalimumab and placebo groups (mean 4860.3 µg/g vs 2981.1 µg/g and 2589.2 µg/g; *P* = .005).

**Table 1.** Baseline characteristics of the study population.

	Overall (n = 300)	Adalimumab (n = 144)	Infliximab (n = 73)	Adalimumab/infliximab (n = 217)	Placebo (n = 83)	*P* value (adalimumab vs infliximab vs placebo)	*P* value (drug vs placebo)
Age, y	38.8 ± 13.0	39.2 ± 12.9	37.2 ± 13.2	38.5 ± 13.0	39.6 ± 12.9	.456	.511
Female	124 (41.3)	58 (40.3)	26 (35.6)	84 (38.7)	40 (48.2)	.264	.136
Disease duration, y	5.9 ± 6.3	5.6 ± 5.8	6.5 ± 6.9	6.1 ± 6.4	6.1 ± 6.4	.564	.833
Disease extent							
Left sided	187 (62.3)	83 (57.6)	69 (94.5)	152 (70.0)	35 (42.2)	.635	.286
Pancolitis	113 (37.7)	61 (42.4)	4 (5.5)	65 (30.0)	48 (57.8)		
Concomitant corticosteroid use	126 (42.0)	62 (43.1)	33 (45.2)	95 (43.8)	31 (37.3)	.636	.672
Concomitant immunomodulator use	96 (32.0)	50 (34.7)	24 (32.9)	74 (34.1)	22 (26.5)	.247	.363
Baseline C-reactive protein, mg/dL	11.0 ± 16.7	12.7 ± 20.2	9.4 ± 12.2	11.6 ± 18.0	9.3 ± 12.6	.222	.298
Baseline fecal ­calprotectin, μg/g	3313.6 ± 4431.1	2981.1 ± 3218.4	4860.3 ± 6466.6	3582.1 ± 4585.7	2589.2 ± 3922.3	.005	.102
Baseline segmental MES (out of 9)	7.0 ± 1.5	7.0 ± 1.5	7.1 ± 1.3	7.0 ± 1.5	6.8 ± 1.7	.482	.271
Baseline descending colon MES (out of 3)	2.3 ± 0.5	2.4 ± 0.5	2.3 ± 0.5	2.4 ± 0.5	2.3 ± 0.5	.239	.683
Baseline sigmoid colon MES (out of 3)	2.4 ± 0.7	2.3 ± 0.7	2.5 ± 0.6	2.4 ± 0.7	2.3 ± 0.7	.654	.636
Baseline rectum MES (out of 3)	2.3 ± 0.8	2.3 ± 0.8	2.3 ± 0.8	2.3 ± 0.8	2.2 ± 0.9	.453	.538
Baseline conventional MES (out of 3)	2.5 ± 0.5	2.5 ± 0.5	2.6 ± 0.5	2.5 ± 0.5	2.5 ± 0.5	.637	.680
Baseline rectal bleeding subscore						.590	.083
0	3 (1.0)	0	3 (4.1)	3 (1.4)	0		
1	87 (29.0)	42 (29.2)	23 (31.5)	65 (30.0)	22 (26.5)		
2	180 (60.0)	90 (62.5)	39 (53.4)	129 (59.5)	51 (61.5)		
3	30 (10.0)	12 (8.3)	8 (11.0)	20 (9.2)	10 (12.1)		
Baseline stool frequency subscore						.760	.787
0	4 (1.3)	1 (0.7)	1 (1.4)	2 (0.9)	2 (2.4)		
1	54 (18.0)	23 (16.0)	17 (23.3)	40 (18.4)	14 (16.9)		
2	119 (39.7)	58 (40.3)	27 (37.0)	85 (39.2)	34 (41.0)		
3	123 (41.0)	62 (43.1)	28 (38.4)	90 (41.5)	33 (39.8)		

Values are mean ± SD or n (%).

Abbreviation: MES, Mayo endoscopic score.

### Segmental patterns of healing

**Figure 1.**
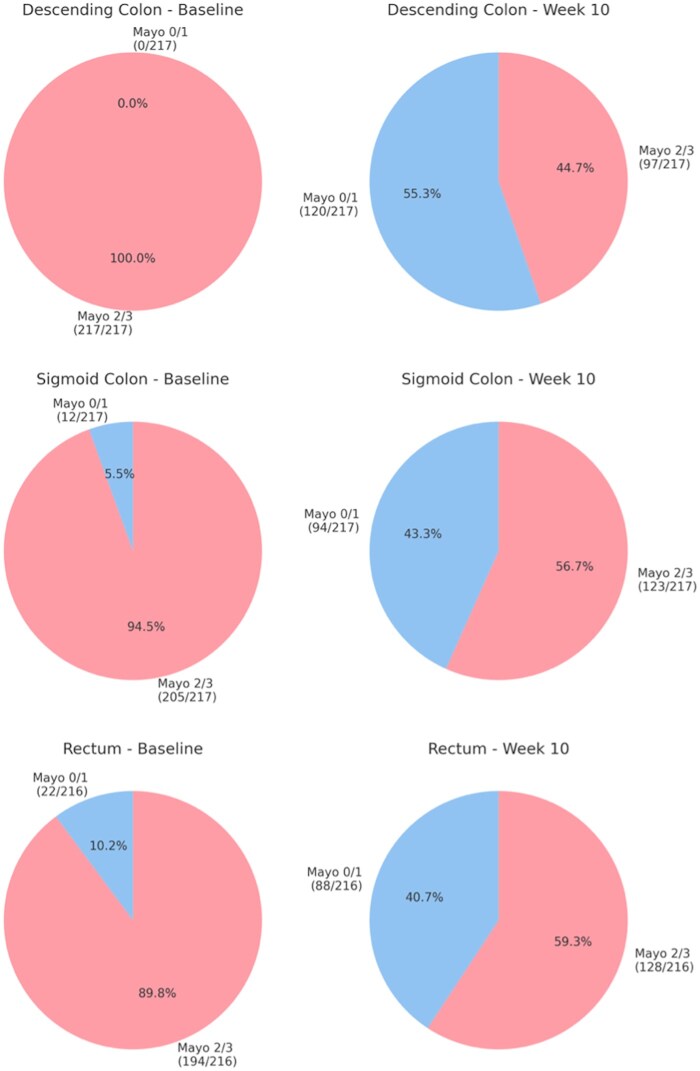
Baseline and week 10 endoscopic scores of the descending colon, sigmoid colon, and rectum among adalimumab- or infliximab-treated participants.

**Figure 2.**
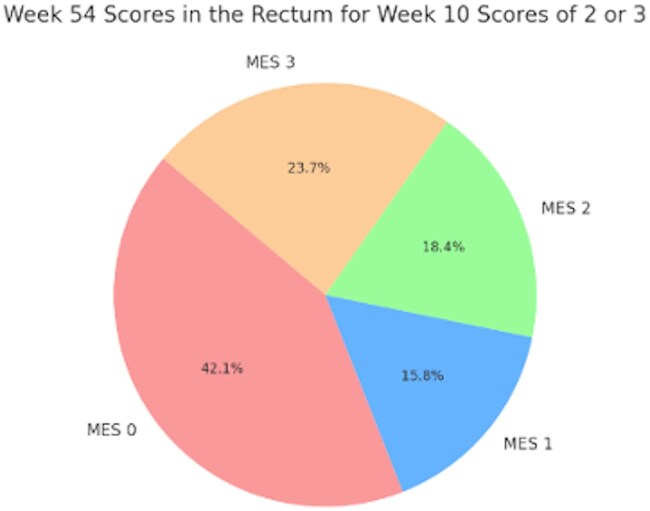
Week 54 endoscopic scores of the rectum among participants treated with infliximab in GARDENIA who had week 10 endoscopic scores of 2 or 3 in the rectum. MES, Mayo endoscopic score.

Segmental analysis of healing among anti-TNF–treated participants revealed that proximal segments (ie, descending colon) showed more rapid improvement compared with distal segments (ie, sigmoid colon and rectum). As demonstrated in Figure 1, by week 10, a greater proportion of anti-TNF–treated participants achieved MES ≤1 in the descending colon (55.3%) compared with the sigmoid colon (43.3%; *P* = .016) and rectum (40.7%; *P* = .003). By week 10, a significantly greater proportion of anti-TNF–treated participants achieved segmental MES 0 in the descending colon (38.3%) compared with placebo (22.9%) (*P* = .013) (Supplemental Table 1). Similar trends were observed in the sigmoid colon and rectum. Further analysis among infliximab-treated patients in GARDENIA demonstrated that the trend for proximal-to-distal healing continues, as 22 (57.9%) of 38 patients who had MES 2 or 3 in the rectum at week 10 were able to attain MES 0 or 1 by week 54 (Figure 2). Sensitivity analyses were performed among participants with MES of 2 or 3 in the sigmoid colon without any involvement in the descending colon (Supplemental Figure 1). Again, by week 10, a greater proportion of anti-TNF–treated participants achieved MES ≤1 in the sigmoid colon as compared with the rectum (48.0% vs 37.6%; *P* = .001).

### Endoscopic outcomes at week 10

By week 10, participants treated with adalimumab or infliximab demonstrated significant improvements in endoscopic outcomes compared with the placebo group. The mean segmental MES was lower in the anti-TNF–treated groups (adalimumab/infliximab combined mean 4.6 ± 3.1) compared with placebo (mean 5.5 ± 2.6) (*P* = .028) (Tables 2 and 3). Declines in segmental MES from baseline were also significantly greater among patients treated with adalimumab/infliximab (mean −35.2 ± 43.5%) compared with placebo (mean −18.5 ± 36.3%) (*P* = .028). Similarly, the conventional MES was significantly lower in the adalimumab/infliximab group (mean 1.7 ± 1.1) compared with placebo (mean 2.1 ± 0.9) (*P* = .001). Declines in conventional MES from baseline were significantly greater among adalimumab/infliximab-treated patients (mean −33.6 ± 43.5%) compared with placebo-treated patients (mean −12.9 ± 35.7%) (*P* < .001).

**Table 2.** Conventional endoscopic outcomes at week 10 among the study population.

Week 10 outcome	Overall (n = 300)	Adalimumab (n = 144)	Infliximab (n = 73)	Adalimumab/infliximab (n = 217)	Placebo (n = 83)	*P* value (adalimumab vs infliximab vs placebo)	*P* value (drug vs placebo)
Conventional MES (out of 3)	1.8 ± 1.1	1.7 ± 1.2	1.6 ± 1.0	1.7 ± 1.1	2.1 ± 0.9	.032	.001
Conventional MES ≤1	109 (36.3)	54 (37.5)	39 (53.4)	93 (42.9)	16 (19.3)	<.001	<.001
Conventional MES of 0	48 (16.0)	33 (22.9)	10 (13.7)	43 (19.8)	5 (6.0)	.003	.004
Percent change in conventional MES	−27.9 ± 42.5	−32.1 ± 45.5	−36.8 ± 39.5	−33.6 ± 43.5	−12.9 ± 35.7	<.001	<.001

Values are mean ± SD or n (%).

Abbreviation: MES, Mayo endoscopic score.

**Table 3.** Segmental endoscopic outcomes at week 10 among the study population.

Week 10 outcome	Overall (n = 300)	Adalimumab (n = 144)	Infliximab (n = 73)	Adalimumab/infliximab (n = 217)	Placebo (n = 83)	*P* value (adalimumab vs infliximab vs placebo)	*P* value (drug vs placebo)
Segmental MES (out of 9)	4.9 ± 3.0	4.7 ± 3.1	4.5 ± 2.9	4.6 ± 3.1	5.5 ± 2.6	.085	.028
Percent change in segmental MES from baseline	−30.6 ± 42.2	−35.2 ± 43.7	−35.2 ± 43.7	−35.2 ± 43.5	−18.5 ± 36.3	.025	.028
Segmental MES ≤3 with no individual segmental score >1	78 (26.0)	42 (29.2)	24 (32.9)	66 (30.4)	12 (14.5)	.016	.005
Segmental MES ≤1 in at least 1 segment that was previously MES 2 or 3 at baseline and MES improvement ≥1 in another segment	131 (43.7)	66 (45.8)	40 (54.8)	106 (48.9)	25 (30.1)	.006	.003
At least 1 segment achieved MES ≤1 without worsening of endoscopic disease in any other segment from baseline	156 (52.0)	76 (52.8)	44 (60.3)	120 (55.3)	36 (43.4)	.105	.064
Segmental MES improvement ≥1 or conventional MES ≤1	204 (68.0)	101 (70.1)	52 (71.2)	153 (70.5)	51 (61.5)	.318	.132
Segmental MES improvement ≥2 or conventional MES ≤1	164 (54.7)	83 (57.6)	47 (64.4)	130 (59.9)	34 (41.0)	.008	.003
Segmental MES improvement ≥3 or conventional MES ≤1	145 (48.3)	72 (50.0)	42 (57.5)	114 (52.5)	31 (37.4)	.036	.019
Segmental MES improvement ≥4 or conventional MES ≤1	124 (41.3)	60 (41.7)	41 (56.2)	101 (46.5)	23 (27.7)	.002	.003
Segmental MES improvement ≥5 or conventional MES ≤1	119 (39.7)	58 (40.3)	40 (54.8)	98 (45.2)	21 (25.3)	.001	.002
Segmental MES improvement ≥6 or conventional MES ≤1	114 (38.0)	56 (38.9)	40 (54.8)	96 (44.2)	18 (21.7)	<.001	<.001
Segmental MES improvement ≥7 or conventional MES ≤1	113 (37.7)	55 (38.2)	40 (54.8)	95 (43.8)	18 (21.7)	<.001	<.001
Segmental MES improvement ≥8 or conventional MES ≤1	112 (37.3)	54 (37.5)	40 (54.8)	94 (43.3)	18 (21.7)	.001	<.001
Segmental MES improvement ≥9 or conventional MES ≤1	111 (37.0)	54 (37.5)	39 (53.2)	93 (42.9)	18 (21.7)	<.001	.001
Segmental MES improvement ≥10%	44 (14.7)	20 (13.9)	10 (13.7)	30 (13.8)	14 (16.9)	.800	.505
Segmental MES improvement ≥20%	26 (8.7)	9 (6.3)	5 (6.9)	14 (6.5)	12 (14.5)	.087	.027
Segmental MES improvement ≥30	8 (2.7)	3 (2.1)	2 (2.7)	5 (2.3)	3 (3.6)	.788	.529
Segmental MES improvement ≥40%	4 (1.3)	2 (1.4)	2 (2.7)	4 (1.8)	0	.329	.213
Segmental MES improvement ≥50%	3 (1.0)	2 (1.4)	1 (1.4)	3 (1.4)	0	.560	.282

Values are mean ± SD or n (%).

Abbreviation: MES, Mayo endoscopic score.

The proportion of participants achieving conventional EI (MES ≤1) and conventional endoscopic remission (MES of 0) was significantly higher in the anti-TNF–treated groups than in the placebo group (42.9% vs 19.3%; *P* < .001; and 19.8% vs 6.0%; *P* = .004, respectively). While other definitions of endoscopic response using segmental MES at week 10 demonstrated significant differences, the conventional EI outcome had the greatest delta in treatment effect between anti-TNF– and placebo-treated patients compared with any other endoscopic outcome definitions assessed herein irrespective of segmental analyses approaches. In terms of segmental MES outcomes, a significantly greater proportion of anti-TNF–treated participants achieved these outcomes at week 10, including segmental MES ≤3 with no individual segmental score >1 (30.4% vs 14.5%; *P* = .005) and segmental MES ≤1 in at least 1 segment that was previously MES 2 or 3 at baseline and MES improvement ≥1 in another segment (48.9% vs 30.1%; *P* = .003). Improvements in segmental MES were also assessed, with significant differences observed between anti-TNF–treated and placebo groups for segmental MES improvement ≥2 or conventional MES ≤1. However, no significant differences between groups were observed for segmental MES improvement based on percent improvement. A greater number of anti-TNF–treated participants achieved MES ≤1 in at least 1 segment without worsening of endoscopic disease in any other segment from baseline, although this did not reach statistical significance.

### Clinical and combined clinical-endoscopic outcomes at week 10

Table 4 demonstrates the proportion of participants achieving clinical outcomes at week 10. Clinical response, defined as a reduction in the total Mayo score by at least 3 points and 30%, with RBS reduction ≥1 or RBS ≤1, was observed in 57% of participants. PRO2 remission (PRO2 score of 0) was observed among 20% of participants. PRO2 response alternatively defined as PRO2 score ≤1 in which RBS was 0 and the stool frequency subscore was ≤1 was achieved among 37.3% of participants. No significant differences between treatment groups were observed for these outcomes, largely due to high response rates among placebo-treated patients. However, a significantly greater proportion of anti-TNF–treated participants achieved a combination of PRO2 response and a conventional MES ≤1 compared with placebo (28.6% vs 13.3%; *P* = .006).

**Table 4.** Clinical outcomes at week 10 among the study population.

	Overall (n = 300)	Adalimumab (n = 144)	Infliximab (n = 73)	Adalimumab/infliximab (n = 217)	Placebo (n = 83)	*P* value (adalimumab vs infliximab vs placebo)	*P* value (drug vs placebo)
Clinical response (reduction in total Mayo score ≥3 points and ≥30% from baseline with an accompanying decrease in RBS of ≥1 point or absolute RBS of ≤1 point) at week 10	171 (57.0)	81 (56.3)	49 (67.1)	130 (59.9)	41 (49.4)	.080	.100
PRO2 normalization (PRO2 score of 0) at week 10	60 (20.0)	29 (20.1)	20 (27.4)	49 (22.6)	11 (13.3)	.088	.071
PRO2 normalization (PRO2 score ≤1 in which RBS = 0 and SFS ≤1) at week 10	112 (37.3)	51 (35.4)	34 (46.6)	85 (39.2)	27 (32.5)	.156	.287
PRO2 ≤1 and MES ≤1	73 (24.3)	36 (25.0)	26 (35.6)	62 (28.6)	11 (13.3)	.005	.006
Clinical response (reduction in total Mayo score ≥3 points and ≥30% from baseline with an accompanying decrease in RBS of ≥1 point or absolute RBS of ≤1 point) among participants with MES ≤1 at week 10	97/114 (82.9)	48/56 (85.7)	35/40 (87.5)	83/96 (86.5)	14/18 (77.8)	.971	.837
PRO2 normalization (PRO2 score of 0) among participants with MES ≤1 at week 10	45/114 (39.5)	22/56 (39.3)	17/40 (42.5)	39/96 (40.6)	6/18 (33.3)	.803	.561
PRO2 normalization (PRO2 score ≤1 in which RBS = 0 and SFS ≤1) among participants with MES ≤1 at week 10	74/114 (64.9)	37/56 (66.1)	26/40 (65.0)	63/96 (65.6)	11/18 (61.1)	.929	.713

Values are mean ± SD, n (%), or n/n (%).

Abbreviations: MES, Mayo endoscopic score; PRO2, Patient-Reported Outcome 2; RBS, rectal bleeding subscore; SFS, stool frequency subscore.

Among 114 participants with a conventional MES ≤1 at week 10, 82.9% (n = 97 of 114) experienced clinical response, 39.5% (n = 45 of 114) achieved PRO2 normalization (PRO2 score of 0) and 64.9% achieved PRO2 normalization alternatively defined as PRO2 score ≤1 in which RBS was 0 and stool frequency subscore was ≤1. Again, no significant differences between treatment groups were observed for these outcomes.

## Discussion

In our study, several key observations were made. First, we found that proximal segments, such as the descending colon, had higher rates of healing compared with distal segments such as the rectum by week 10. Based on our analysis, this pattern of healing continues to move proximally to distally as over half of participants in GARDENIA with MES of 2 or 3 in the rectum at week 10 had improvement of the rectum by week 54. This aligns with prior hypotheses that proximal segments may respond more rapidly to treatment due to differences in disease pathophysiology or mucosal characteristics.^13^ These findings also suggest that segmental evaluation of disease could be used as an adjunctive strategy to assess endoscopic burden in clinical trials for UC, and may help to select patients who are having improvement in their endoscopic burden of disease despite having a worst-affected area of MES 2 or 3 still present at the completion of induction therapy.

It is widely believed that in UC, disease extension occurs from distal to proximal colon,^14^ and that healing occurs in an opposite fashion from proximal to distal; however, no published studies to date have revealed endoscopic patterns of healing in UC by anatomical location within the colon. This study demonstrates segmental patterns of endoscopic healing in UC and highlights the utility of granular endoscopic scoring systems for evaluating treatment efficacy in specific segments of the colon. Our findings demonstrate that in moderate-severe UC, healing follows a proximal-to-distal pattern, with the descending colon demonstrating higher rates of healing by week 10 compared with the sigmoid colon and rectum. We also did not find enhanced detection of treatment effect when using a segmental scoring approach as compared with conventionally measured MES.

The ability to detect early healing is important for clinical trials evaluating novel therapies for UC. Prior to their approval, novel therapies must demonstrate the ability to heal the colonic mucosa as part of primary or co-primary endpoints in placebo-controlled clinical trials. Although segmental MES offered a more nuanced understanding of endoscopic disease burden and the expected pattern of healing, we demonstrated that EI as defined by conventional MES ≤1 had the greatest delta at week 10 compared with any other definition of EI assessed, including improvements in segmental MES. This was likely due to the segmental approach being limited by very high response rates in placebo-treated patients, which diluted the ability to detect differences in treatment effects between anti-TNF– and placebo-treated groups. This highlights the challenge of utilizing more granular scoring systems in settings in which placebo response is substantial and obscures the incremental benefits of active therapy.^15^ Therefore, our findings suggest continued use of the conventionally scored MES for assessment of endoscopic burden in UC. However, there may be other approaches in which measuring endoscopic burden beyond worst-affected area as accomplished by the MES helps to enhance detection of treatment effect. For example, a post hoc analysis of endoscopic data from UNIFI demonstrated that spatial mapping of the MES, termed the Cumulative Disease Score, reduced the sample size needed to detect endoscopic differences between ustekinumab and placebo by 50%. Compared with the MES, the Cumulative Disease Score demonstrated superior sensitivity to treatment effects as it is the sum score of MES-squared values of 50 evenly spaced increments in the left colon and rectum, accounting for the total endoscopic disease burden.^10^ Future investigation is warranted to determine if segmental scoring can enhance the detection of treatment response when used with more granular endoscopic indices. These indices, such as the Ulcerative Colitis Endoscopic Index of Severity and the Ulcerative Colitis Colonoscopic Index of Severity, provide detailed assessments of ulcer depth and extent.^16^^,^^17^

Beyond EI, novel therapies are required to demonstrate improvement in symptoms within clinical trials in order to gain regulatory approval. However, there continues to be debate about the best ways to measure clinical symptom improvement, and in particular whether relying on scoring systems that incorporate more subjective measures such as symptoms or physician global assessment could lessen detection of treatment effect in from potentially effective therapies.^14^^,^^18^ Our study found that reliance upon PRO2 normalization (PRO2 ≤1) alone did a poor job at distinguishing between anti-TNF– and placebo-treated patients at week 10 (39.2% vs 32.5%; *P* = .287). However, assessing for EI in addition to PRO2 normalization enhanced detection of treatment effect between anti-TNF– and placebo-treated patients (anti-TNF–treated vs placebo 28.6% vs 13.3%; *P* = .006). The limitations of relying solely on PRO2 scores are evident in their reduced sensitivity to treatment-­specific effects. PRO2 normalization often focuses on patient-­reported outcomes like rectal bleeding, and stool frequency, which, while important, may not fully reflect the degree of mucosal healing achieved. By contrast, the MES provides an objective measure of EI, which is a critical component of long-term disease control and a predictor of favorable outcomes such as sustained remission and reduced hospitalization rates. Combining PRO2 and MES ensures a more robust evaluation of therapeutic benefit, which could guide clinicians in selecting optimal treatments for patients. From a clinical perspective, the study emphasizes the importance of integrating endoscopic outcomes with clinical response measures, such as PRO2 normalization, to fully capture therapeutic benefits. Participants achieving a combination of PRO2 normalization and MES ≤1 were significantly more common in the treatment groups, reinforcing the role of biologics in achieving both symptomatic relief and mucosal healing.

Previously, Christensen et al^19^ demonstrated that most patients with UC (88%) experienced histologic healing in a proximal-to-distal direction, with segmental histological normalization occurring in one-third of participants. However, segmental histological normalization was not associated with improved clinical outcomes based on PRO2 normalization in their study. Moreover, this study did not identify a proximal-to-distal healing pattern. While histology was not assessed in our study, given the correlation between histologic and endoscopic disease severity, the lack of association between segmental histological normalization and PRO2-based outcomes reinforces our finding that relying solely on PROs to gauge disease improvement is insufficient compared with additional of more objective measures such as endoscopy or histology.

This study has several notable strengths. First, it leverages individual participant-level data from multiple randomized, placebo-controlled clinical trials. The use of both conventional and segmental MES allowed for a detailed exploration of mucosal healing patterns, highlighting differences in healing dynamics across colonic segments. Furthermore, our study provides valuable insights into the added utility of combined clinical and endoscopic endpoints, which better capture treatment effects compared with isolated measures like PRO2 normalization. These findings have important implications for both clinical practice and UC trials. The observed proximal-to-distal healing pattern suggests that clinicians should be cautious when making decisions about treatment efficacy based solely on early follow-up of the distal colon. For example, persistent rectal inflammation at week 10 may not signify an overall treatment failure if more proximal segments are showing EI. This could inform the timing of decisions about switching therapies, potentially preventing premature discontinuation of an effective treatment. Furthermore, these findings support the utility of performing a more extensive endoscopic evaluation (eg, flexible sigmoidoscopy to at least the splenic flexure) to accurately assess the full extent of mucosal healing, rather than relying on a limited distal assessment. Our study was restricted to patients with active disease up to the descending colon and therefore cannot definitively conclude whether this healing pattern applies to more proximal segments in extensive colitis (E3 disease), or on whether certain segments heal quicker than others. Future studies focusing on patients with pancolitis are needed to clarify these patterns.

However, several limitations are worth noting. As a post hoc analysis, our results are inherently exploratory and require validation in prospective studies. Additionally, the follow-up period of 10 weeks may not fully capture the long-term effects of biologic therapies on mucosal healing and clinical outcomes. The study population, drawn from clinical trials, may also differ from real-world UC populations in terms of disease severity, comorbidities, and treatment history, potentially limiting generalizability. Specifically, we restricted our analyses to treatment-naïve patients with an MES of 2 or 3 in the descending colon. We were only able to assess patients treated with adalimumab or infliximab against placebo, and while both biologics showed comparable efficacy in most outcomes, it is worth noting that infliximab-treated participants demonstrated slightly higher proportions of complete mucosal healing (MES = 0) and segmental MES improvement. These observations, while not statistically significant, may warrant further investigation into potential differences in efficacy profiles between these agents. In addition, while the segmental MES provided greater granularity, it introduces complexity that may not be practical for routine clinical use. Last, we did not assess variables associated with proximal healing given the skew of our study population toward those with left-sided disease.

In conclusion, this study confirmed a proximal-to-distal pattern of healing in UC. Although EI as measured conventionally demonstrated the largest treatment effect between biologic- and placebo-treated patients in this study, there may be utility for measurement of endoscopic burden beyond conventional MES scoring. Future studies should address these limitations by exploring segmental MES in diverse, real-world cohorts treated with a wider variety of biologics and followed over longer durations to explore how segmental MES improvements correlate with long-term outcomes, such as relapse prevention and sustained remission.

## Supplementary Material

izaf199_Supplementary_Data

## Data Availability

This publication (Vivli protocol #00010343) is based on research using data from Roche Inc that has been made available through Vivli, Inc. Vivli has not contributed to or approved, and is not in any way responsible for, the contents of this publication. Data can be made available upon request.
