# Supplementary material for: Anti-TNF Therapies Promote a Proximal-to-Distal Healing Pattern in Moderate-to-Severe Ulcerative Colitis
Source: Inflamm Bowel Dis. 2025 Sep 25;32(1):87–96. doi: 10.1093/ibd/izaf199 (PMC12759055; doi:10.1093/ibd/izaf199)
Supplement: izaf199_Supplementary_Data [file izaf199_supplementary_data.zip › Supplementary Appendix.docx]

Supplementary Table 1 – Baseline and week 10 endoscopic scores among participants

|  | Overall (n=300) | Adalimumab (n=144) | Infliximab (n=73) | Ada/Ifx (n=217) | Placebo (n=83) | p-value (Ada vs. ifx vs. placebo) | p-value (Drug vs. Placebo) |
| --- | --- | --- | --- | --- | --- | --- | --- |
| Baseline MES of the descending colon, n (%)  2  3 | 198 (66.0) 102 (34.0) | 91 (63.2) 53 (36.8) | 50 (68.5) 23 (31.5) | 141 (65.0) 76 (35.0) | 57 (55.3) 26 (25.2) | 0.615 | 0.545 |
| Baseline MES of the sigmoid colon, n (%)  0  1  2  3 | 8 (2.7) 10 (3.3) 143 (47.7) 139 (46.3) | 3 (2.1) 7 (4.9) 71 (49.3) 63 (43.8) | 1 (1.4) 7 (4.9) 71 (49.3) 63 (43.8) | 4 (1.8) 8 (3.7) 101 (46.5) 104 (47.9) | 4 (4.8) 2 (2.4) 42 (50.6) 35 (42.2) | 0.313 | 0.403 |
| Baseline MES of the rectum, n (%)*  0  1  2  3 | 17 (5.7) 19 (6.4) 130 (43.6) 132 (44.3) | 8 (5.6) 7 (4.9) 65 (45.1) 64 (44.4) | 4 (5.6) 3 (4.2) 33 (45.8) 32 (44.4) | 12 (5.6) 10 (4.6) 98 (45.4) 96 (44.4) | 5 (6.1) 9 (11.0) 32 (39.0) 36 (43.9) | 0.626 | 0.227 |
| Week 10 MES of the descending colon, n (%)  0  1  2  3 | 102 (34.0) 49 (16.3) 91 (30.3) 58 (19.3) | 55 (38.2) 21 (14.6) 38 (26.4) 30 (20.8) | 28 (38.4) 16 (21.9) 17 (23.3) 12 (16.4) | 83 (38.3) 37 (17.1) 55 (25.4) 42 (19.4) | 19 (22.9) 12 (14.5) 36 (43.4) 16 (19.3) | 0.042 | 0.013 |
| Week 10 MES of the sigmoid colon, n (%)  0  1  2  3 | 68 (22.7) 47 (15.7) 87 (29.0) 98 (32.7) | 40 (27.8) 18 (12.5) 36 (25.0) 50 (34.7) | 15 (20.6) 21 (28.8) 17 (23.3) 20 (27.4) | 55 (25.4) 39 (18.0) 53 (24.4) 70 (32.3) | 13 (15.7) 8 (9.6) 34 (41.0) 28 (33.7) | 0.002 | 0.013 |
| Week 10 MES of the rectum, n (%)**  0  1  2  3 | 63 (21.1) 53 (17.7) 70 (23.4) 113 (37.8) | 38 (26.6) 20 (14.0) 33 (23.1) 52 (36.4) | 17 (23.3) 13 (17.8) 13 (17.8) 30 (41.1) | 55 (25.5) 33 (15.3) 46 (21.3) 82 (38.0) | 8 (9.6) 20 (24.1) 24 (28.9) 31 (37.4) | 0.047 | 0.010 |

*n=298
**n=299


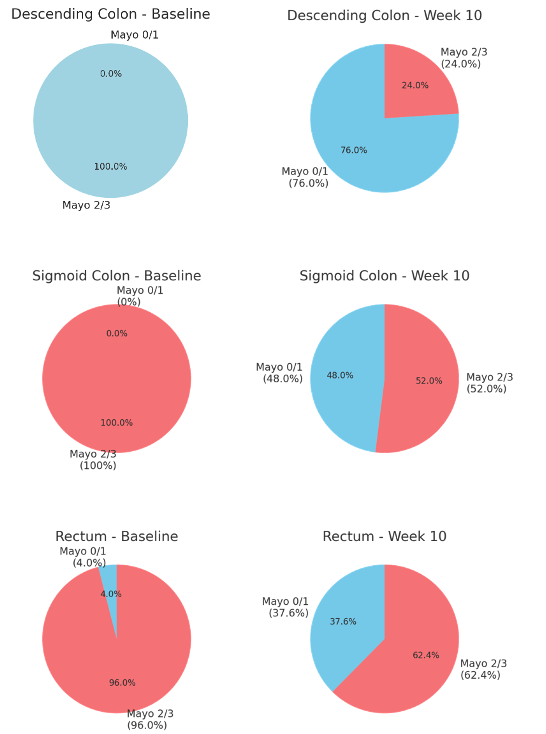
Supplementary Figure 1 – Baseline and week 10 endoscopic scores of the sigmoid colon and rectum among adalimumab or infliximab-treated participants with MES 2 or 3 in the sigmoid (without descending colon involvement) at baseline
